# JNK3 Overexpression in the Entorhinal Cortex Impacts on the Hippocampus and Induces Cognitive Deficiencies and Tau Misfolding

**DOI:** 10.1021/acschemneuro.3c00092

**Published:** 2023-05-26

**Authors:** Carlos G. Ardanaz, Amaia Ezkurdia, Arantza Bejarano, Beatriz Echarte, Cristian Smerdou, Eva Martisova, Iván Martínez-Valbuena, María-Rosario Luquin, María J. Ramírez, Maite Solas

**Affiliations:** † Department of Pharmacology and Toxicology, University of Navarra, 31008 Pamplona, Spain; ‡ IdISNA, Navarra Institute for Health Research, 31008 Pamplona, Spain; § Division of Gene Therapy and Regulation of Gene Expression, Cima Universidad de Navarra, 31008 Pamplona, Spain; ∥ Neurosciences Division, Cima Universidad de Navarra, 31008 Pamplona, Spain; ⊥ Tanz Centre for Research in Neurodegenerative Diseases, University of Toronto, M5S 1A8 Toronto, Canada; # Neurology Department, Clinica Universidad de Navarra, 31008 Pamplona, Spain

**Keywords:** entorhinal cortex, hippocampus, cognitive impairment, Tau, neuroinflammation, gliosis

## Abstract

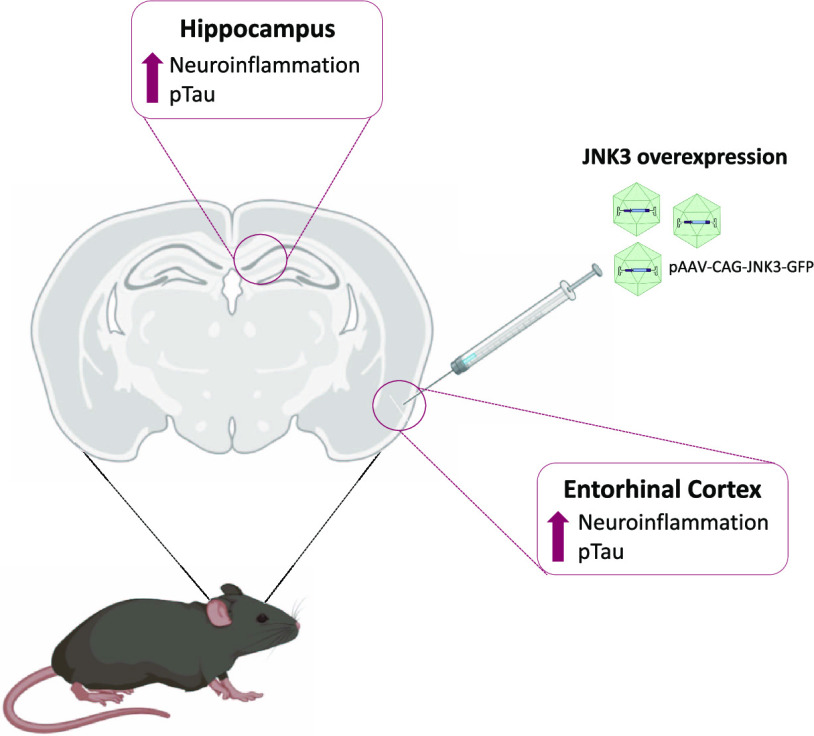

c-Jun N-terminal kinases (JNKs) are a family of protein kinases activated by a myriad of stimuli consequently modulating a vast range of biological processes. In human postmortem brain samples affected with Alzheimer′s disease (AD), JNK overactivation has been described; however, its role in AD onset and progression is still under debate. One of the earliest affected areas in the pathology is the entorhinal cortex (EC). Noteworthy, the deterioration of the projection from EC to hippocampus (Hp) point toward the idea that the connection between EC and Hp is lost in AD. Thus, the main objective of the present work is to address if JNK3 overexpression in the EC could impact on the hippocampus, inducing cognitive deficits. Data obtained in the present work suggest that JNK3 overexpression in the EC influences the Hp leading to cognitive impairment. Moreover, proinflammatory cytokine expression and Tau immunoreactivity were increased both in the EC and in the Hp. Therefore, activation of inflammatory signaling and induction of Tau aberrant misfolding caused by JNK3 could be responsible for the observed cognitive impairment. Altogether, JNK3 overexpression in the EC may impact on the Hp inducing cognitive dysfunction and underlie the alterations observed in AD.

## 1 Introduction

Cells constantly interact with their environment by receiving and sending signals. These cues that the cell receive control many functional aspects by activating different signaling pathways, such as the mitogen-activated kinases called MAPKs (mitogen-activated protein kinases) that are subdivided into three families: p38, ERK, and c-Jun N-terminal kinases (JNKs).^1,2^

The JNK family proteins can be activated by numerous stimuli. When activated, they in turn modify the activity of other proteins by adding phosphate groups.^3^ In this way, JNK regulates important functions in a broad spectrum of biological processes in the cytoplasm, mitochondria, and also in the nucleus, especially in the central nervous system (CNS).^1^

JNK is encoded by three genes, namely, *Jnk1* (also known as *Mapk8*), *Jnk2* (*Mapk9*), and *Jnk3* (*Mapk10*),^4,5^ but due to the alternative splicing, 10 different splice variants can be generated.^1,6,7^ The 10 different variants are grouped depending on the homologous protein regions in the three known isoforms of JNK: JNK1, JNK2, and JNK3. Albeit JNK1 and JNK2 are widely distributed throughout the different tissues, JNK3 is principally found in the CNS.^8^

JNK3 is responsible for regulating the functions of the brain in both healthy and pathological conditions. JNK3 is involved in brain maturing,^9^ neurite creation, and flexibility,^10,11^ and it is implicated in memory capacity and learning.^12,13^ In pathological circumstances, JNK3 has been proposed as a deleterious transducer signal, and it seems to be overstimulated in the adult brain after pernicious stress stimuli, like hypoxia, ischemia, or epilepsies.^8,14−18^

Neuroinflammation is a defense mechanism of the brain, initiated in the CNS by the immune system to protect it from infections and other threats. However, when it becomes chronic, it produces metabolic changes that lead to tissue and cognitive degeneration potentially resulting in pathologies such as Parkinson′s disease (PD), Alzheimer′s disease (AD), and others.^19,20^ It has been reported that the total amount of nuclear JNK is rapidly and transiently increased after a neuroinflammatory stimulus, leading to augmented levels of inducible NO synthase (iNOS) and proinflammatory mediators such as interleukins.^21−23^ These findings indicate that JNK plays crucial roles in the neuroinflammatory processes underlying various neurodegenerative disorders.

AD is a progressive CNS degenerative disease characterized by neurofibrillary tangles^24^ and amyloid-β (Aβ) deposits.^25^ It has been shown that postmortem brains of patients with this disease exhibit anomalously elevated concentrations of JNK activity,^26−28^ and preclinical research using animal models evinces that JNK can have a significant impact on AD pathology increasing Aβ plaque load^28,29^ and Tau hyperphosphorylation.^17,30^ AD mouse models carrying the Swedish mutation on the amyloid precursor protein (APP) and/or a mutant presenilin 1 show increased JNK activity.^28,31,32^ A marked decreased degeneration of pyramidal neurons has been observed when the AD mouse model brain slices has been treated with JNK inhibitors,^33^ and the chronic administration of a JNK inhibitor to an AD mouse model restores memory impairment and LTP abnormalities.^34^ Furthermore, a very elegant study showed that genetic deletion of *Jnk3* in AD mice decreases Aβ plaque burden.^28^

Aside from glycogen synthase kinase 3 (GSK3), p38, and ERK, Tau could be phosphorylated by JNK on various locations that are hyperphosphorylated in paired helical fragments.^30,35^ Patients with AD have shown incremented activity of JNK in neurofibrillary tangles in brain tissue.^36^ In addition, JNK activity is enhanced in tangles in Tg2576/PS1P264L and traumatic brain injury mouse models, where JNK is colocalized with phosphorylated Tau.^31,32^ Noteworthy, D-JNKI-1, which is a JNK inhibitor peptide, reduces Tau phosphorylation and subsequent aggregation.^32^

Entorhinal cortex (EC) is one of the first regions undergoing neuronal cell loss in AD.^37,38^ In both primates and rodents, the EC is located in the temporal lobe and nearby the hippocampus (Hp). Two major divisions can be distinguished: the medial EC (MEC) and the lateral EC (LEC). The EC innervates the Hp through the perforant pathway projection. Indeed, in early phases of AD, the loss of the projection from EC to Hp has led to the hypothesis that the connection between EC and Hp could be degenerated in AD,^38,39^ leading to cognitive deficiencies.

Taking into account the relationship between JNK3, neuroinflammation, hyperphosphorylation of tau, and AD, the present work aims to explore whether an overexpression of JNK3 in the EC could impact on the hippocampus, inducing cognitive deficiencies, similar to that observed in early phases of AD.

## 2 Results

### 2.1 Analysis of AAV Transduction Efficacy

**Figure 1:**
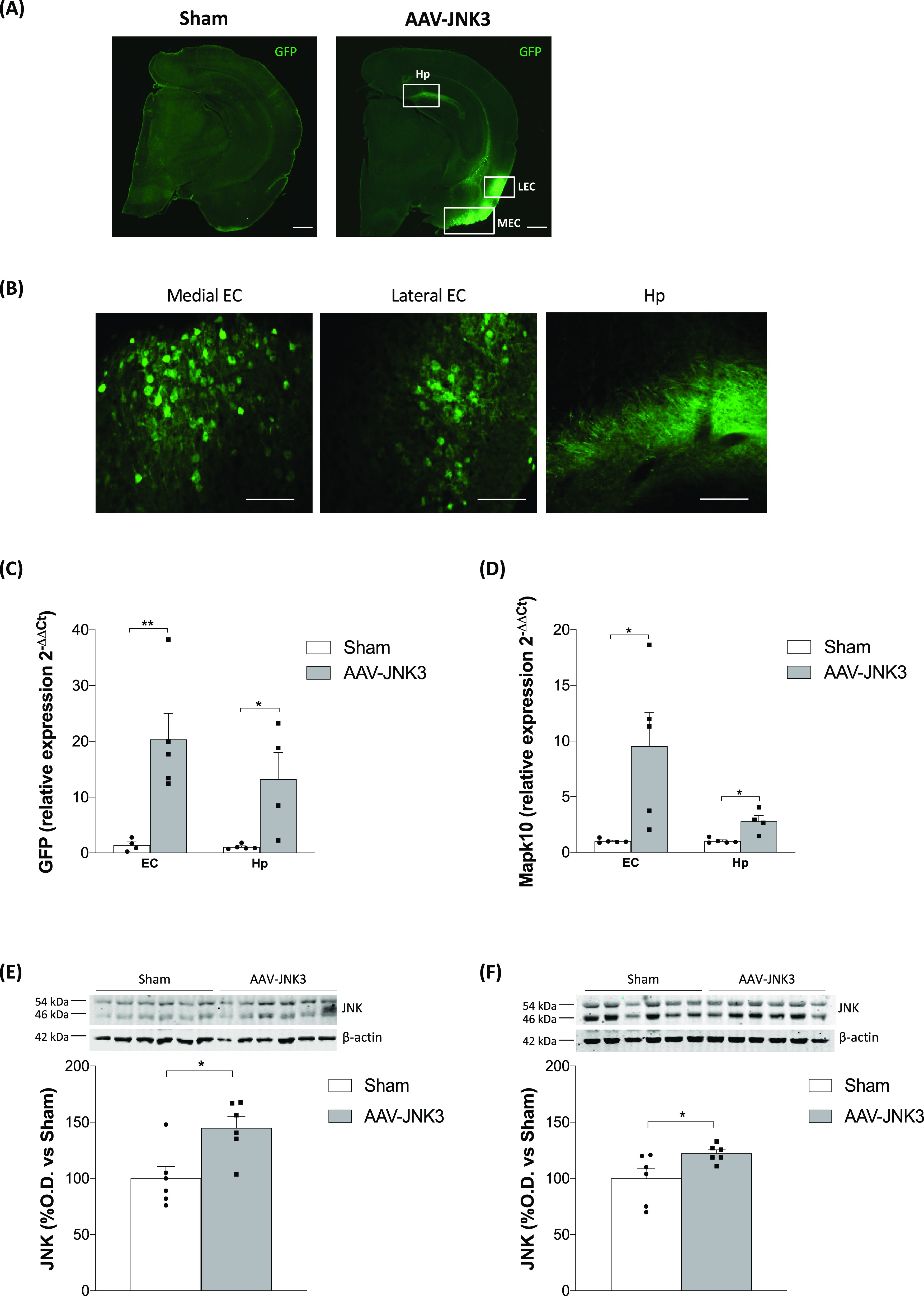
Analysis of the transduction efficacy of the AAV. (A) Representative images of GFP expression in Sham- and AAV-JNK3-injected mice. White boxes indicate key areas magnified in Figure 1B. Scale bar: 1 mm. (B) GFP expression in the medial EC, lateral EC, and Hp. Scale bar: 100 μm. (C) GFP mRNA relative expression in EC (Student′s *t* test, *t* = 3.552, **p < 0.01; *n* = 5) and Hp (Student′s *t* test, *t* = 2.887, *p < 0.05; *n* = 5). (D) MAPK10 mRNA relative expression in the EC (Student′s *t* test, *t* = 2,825, *p < 0.05; *n* = 5) and Hp (Student′s *t* test, *t* = 3.670, *p < 0.05; *n* = 5). (E) JNK protein presence in EC (Student′s *t* test, *t* = 3.110, *p < 0.05; *n* = 6). (F) JNK protein presence in Hp (Student′s *t* test, *t* = 2.315, *p < 0.05; *n* = 6). Results are shown as mean ± SEM. In panels (E) and (F), figures show optical density (O.D.) value percentage and an illustrative image of the blotting. EC: entorhinal cortex; Hp: hippocampus; and O.D.: optical density.

After demonstrating a successful JNK3 expression *in vitro* (Figure S1), a dose of 1 × 10^10^ VG of AAV8-JNK3-GFP vector (AAV-JNK3 group) or PBS (Sham group) was injected bilaterally into the medial and lateral EC by stereotactic injection *in vivo*. Intracranial surgery did not induce any adverse reaction in mice. Three months after viral vector inoculation, mice were sacrificed and GFP expression was analyzed. GFP expression was detected in the targeted area of all AAV-JNK3-treated mice, but no fluorescence was observed in the Sham group (Figure 1A). Neurons in the EC interact extensively with hippocampal neurons, a key brain area that features pathological signs and abundant amyloid plaques in AD. Due to the innervation of the Hp through the perforant pathway projection coming from the EC, we found that some hippocampal areas of AAV-JNK3-treated mice, which seem to correspond with the molecular layer of the dentate gyrus, expressed GFP (Figure 1A). A closer analysis of these areas revealed that while somatic-like fluorescent shapes are observed in the injection site at the EC (Figure 1B, medial EC and lateral EC panels and Figure S2A), fiberlike fluorescent shapes are observed in the Hp (Figure 1B, Hp panel and Figure S2B), which could indicate that the AAV delivered into the EC reaches the Hp through EC axonal projections.

To further demonstrate the viral expression of GFP and JNK3 in the EC and Hp of the AAV-injected mice, qPCRs were performed three months after the injections and compared to similar qPCRs conducted with Sham-injected mice tissue. Our data showed a significant increase of GFP not only in the EC but also in the Hp (Figure 1C). In a similar way, JNK3 expression was markedly increased in the EC and the Hp in the AAV-injected group (Figure 1D).

Western blot analysis of EC and Hp protein extracts obtained from Sham- or AAV-injected mice euthanized three months after injection revealed that entorhinal AAV-JNK3 administration resulted in a significant accumulation of JNK protein compared to Sham-injected animals not only in the injection site, i.e., EC (Figure 1E), but also in the Hp (Figure 1F) and that this accumulation was still present after 3 months.

### 2.2 Behavioral Consequences of JNK3 Overexpression in the EC

**Figure 2:**
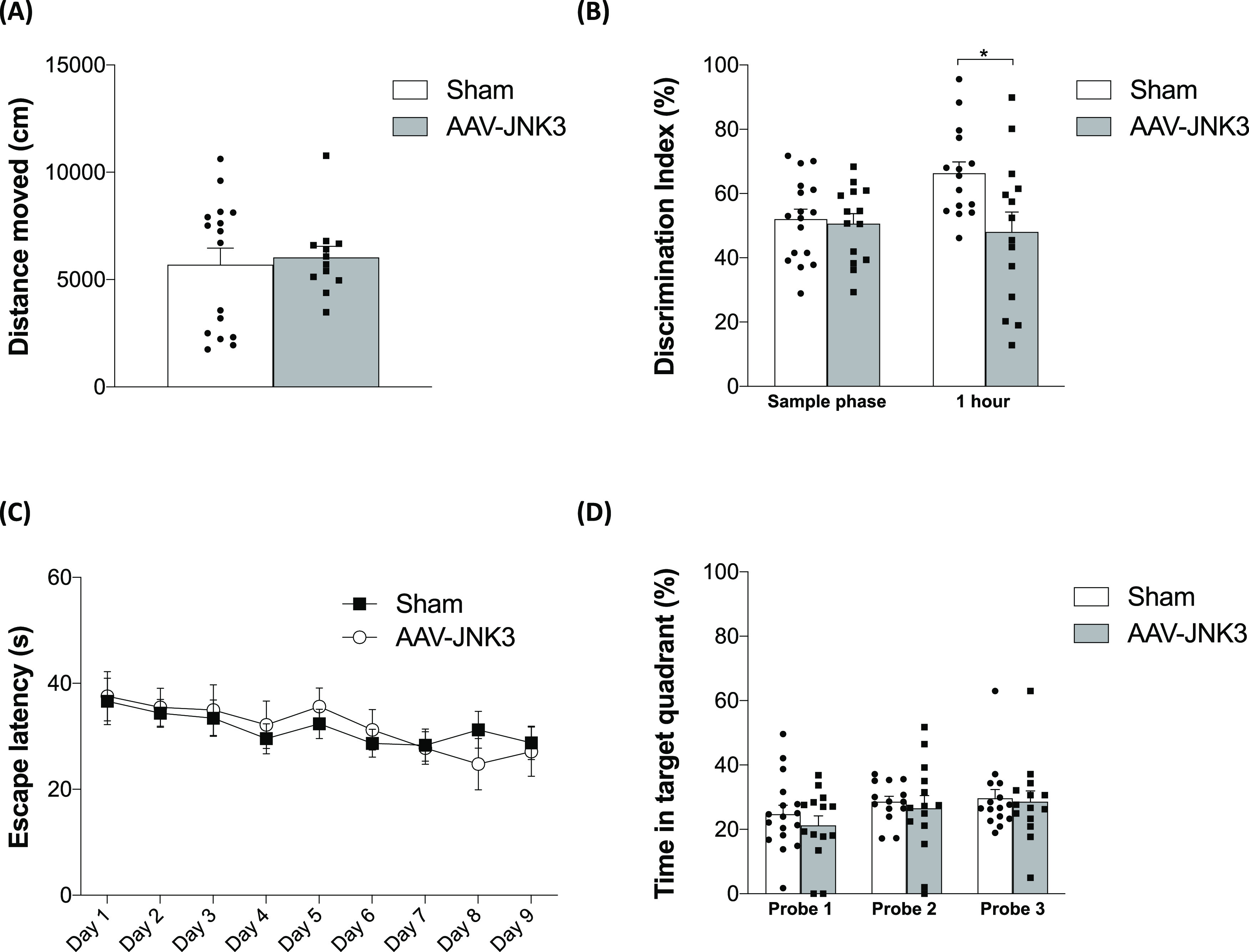
Behavioral consequences of JNK3 overexpression in the EC. (A) Locomotor activity (*n* = 14–15). (B) Cognitive performance in the novel object recognition test (NORT). Data display discrimination index (time exploring the new object/total exploration time × 100) (Student′s *t* test, *t* = 2.582, *p < 0.05; *n* = 14–15). (C) and (D) cognitive performance assessed by the Morris water maze (MWM) acquisition phase and retention phase, respectively (*n* = 14–15). Data are showed as mean ± SEM. EC: entorhinal cortex.

The memory performance of AAV-JNK3-treated mice was assessed using the novel object recognition test (NORT) and the Morris water maze (MWM) paradigm, three months after the AAV injection. Of note, no differences were observed in the locomotor activity between groups, indicating that behavioral capacity differences between Sham- and AAV-injected mice are not due to locomotor activity alterations (Figure 2A).

In the NORT, the percentage of time that mice invested exploring the new object against the old one (discrimination index) was the parameter used to evaluate cognitive performance. As shown in Figure 2B, AAV-JNK3 mice displayed cognitive deficits in the NORT, as indicated by a significantly decreased discrimination index, failing to distinguish between an old and a novel object one hour after exposure to the old object.

In the MWM test, the lack of differences observed among the groups in the escape latency during the visible platform phase indicates that all of the animals are able to perform the task (data not shown). Moreover, swimming speed did not differ between groups (data not shown). As shown in Figure 2C, no significant differences were observed during the invisible platform phase. The memory retention was evaluated at the beginning of the fourth (Probe 1), seventh (Probe 2), and tenth day (Probe 3), and no significant differences were observed in any of those probe trials (Figure 2D), in parallel with the results obtained in the acquisition phase.

### 2.3 Effects of JNK3 Overexpression on Gliosis and Neuroinflammation

It has been suggested that sustained glial activation is a key factor contributing to cognitive impairment and that activation of JNK results in neuroinflammation and subsequent neurodegeneration. Thus, the impact of JNK3 overexpression on glial reactivity and markers of neuroinflammation was studied.

**Figure 3:**
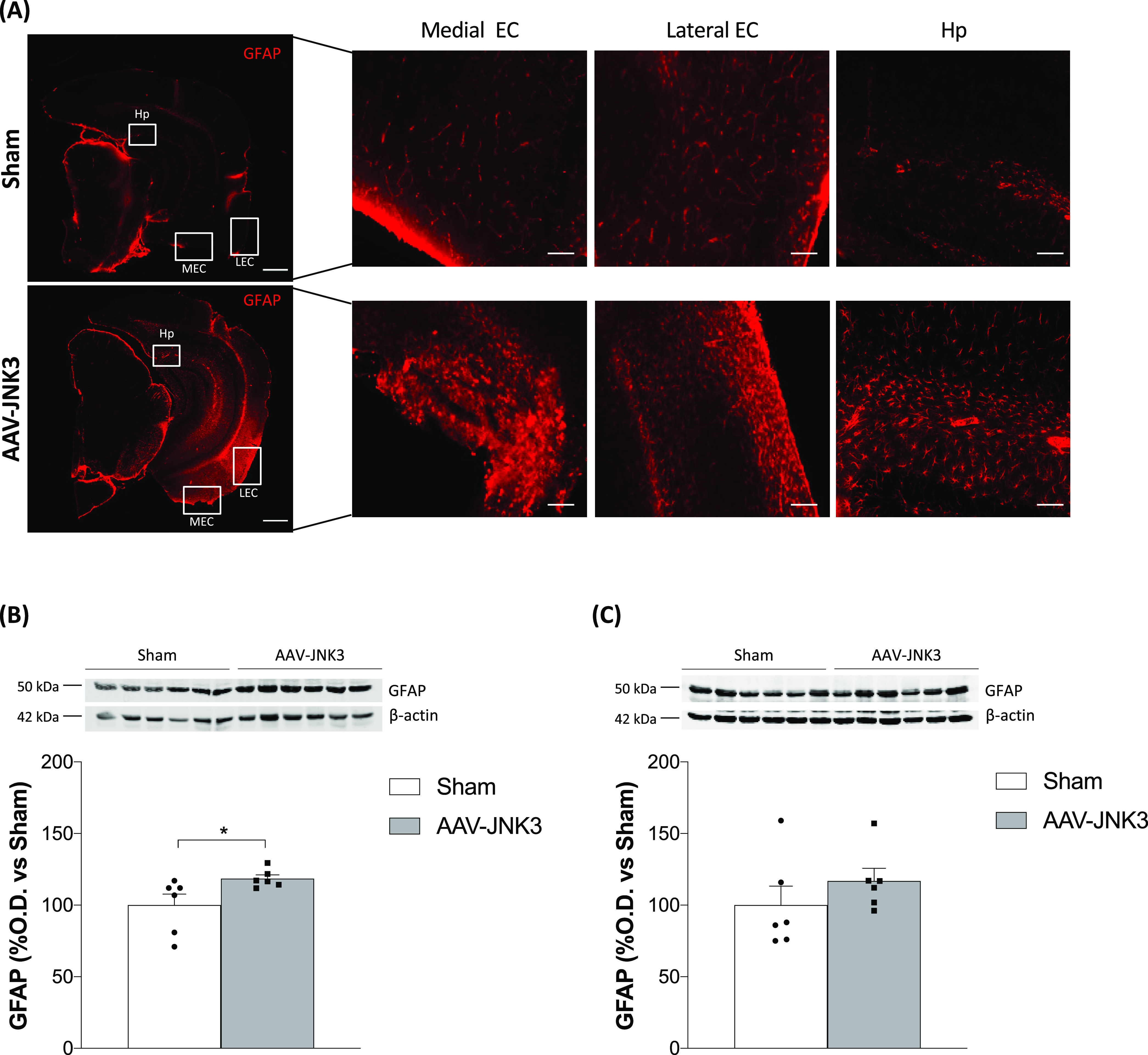
Effects of JNK3 overexpression on astrogliosis. (A) GFAP expression in Sham- and AAV-JNK3-injected mice (scale bar: 1 mm) and magnification images (scale bar: 100 μm) of medial EC, lateral EC, and Hp. (B) GFAP protein levels in the EC (Student′s *t* test, *t* = 2.256, *p < 0.05; *n* = 6). (C) GFAP protein levels in the Hp (Student′s *t* test, *t* = 1.065, *p* > 0.05; *n* = 6). Results are shown as mean ± SEM. In panels (B) and (C), figures show density (O.D.) value percentage and an illustrative image of the blotting. EC: entorhinal cortex; Hp: hippocampus; and O.D.: optical density.

To address astrogliosis, we focused on GFAP, a major intermediate filament protein specific to astrocytes. Our data showed a significant elevation in GFAP immunoreactivity in AAV-JNK3-treated mice compared to Sham mice (Figure 3A), not only in the injected area, i.e., the EC (Figure 3A, medial EC and lateral EC panels), but also in the projection site, i.e., the Hp (Figure 3A, Hp panel). When protein levels were assessed by immunoblotting, a marked increase was observed in the EC (Figure 3B); however, the increase observed in the Hp did not reach a statistical significance (Figure 3C).

**Figure 4:**
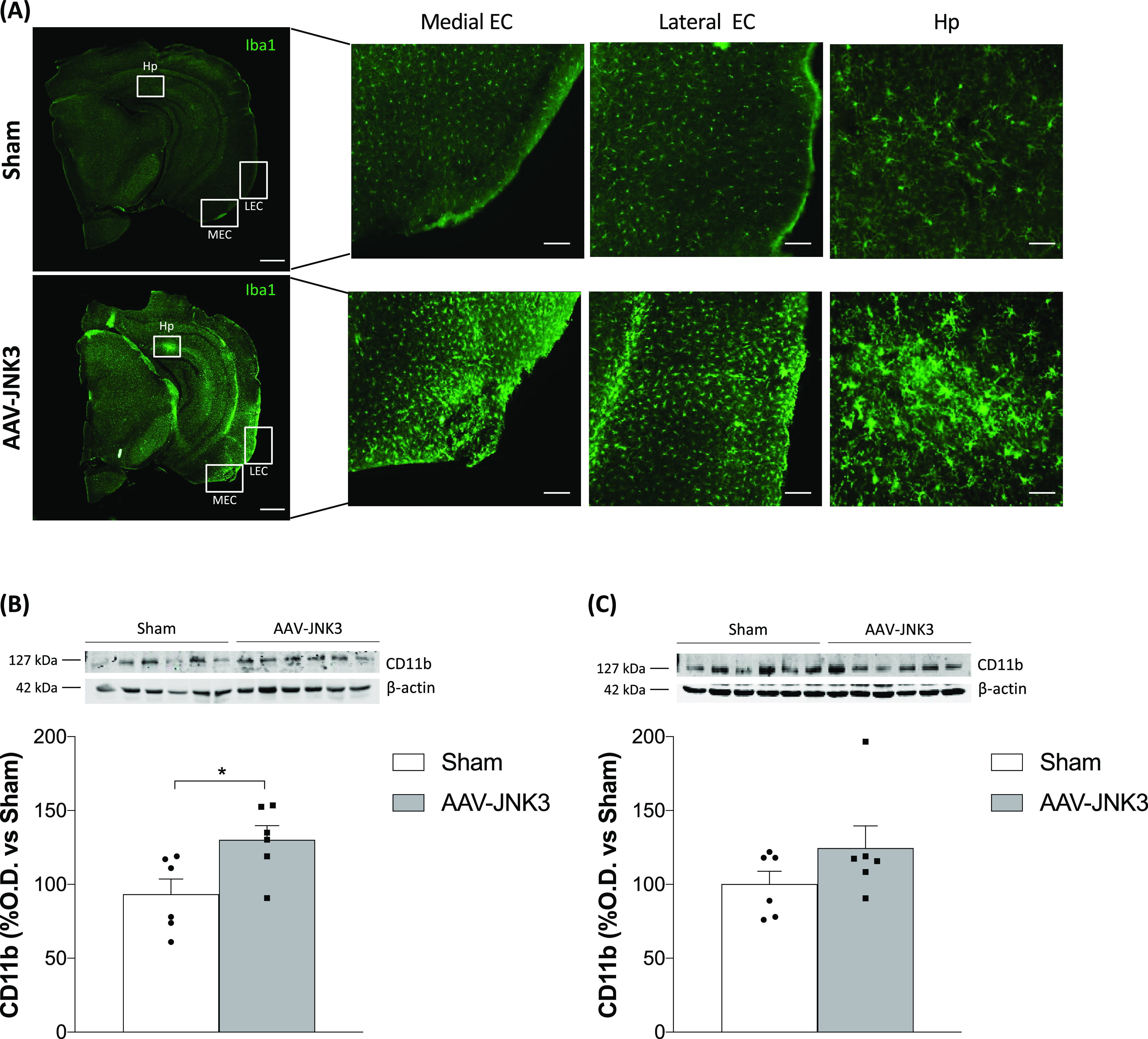
Effects of JNK3 overexpression on microgliosis. (A) Iba1 expression in Sham- and AAV-JNK3-injected mice (scale bar: 1 mm) and magnification images (scale bar: 100 μm) of medial EC, lateral EC, and Hp. (B) CD11b protein levels in the EC (Student′s *t* test, *t* = 2.619, *p < 0.05; *n* = 6). (C) CD11b protein levels in the Hp (Student′s *t* test, *t* = 1.397, *p* > 0.05; *n* = 6). Results are shown as mean ± SEM. In panels (B) and (C), figures show optical density (O.D.) value percentage and an illustrative image of the blotting. EC: entorhinal cortex; Hp: hippocampus; and O.D.: optical density.

To address microgliosis, we focused on Iba1 for immunohistochemistry and CD11b for immunoblotting. In parallel with GFAP, Iba1 immunoreactivity was increased in the EC (Figure 4A, medial EC and lateral EC panels), as well as in the Hp (Figure 4A, Hp panel). Again, when measured by immunoblotting, while a marked increase was observed in the EC (Figure 4B), the increase in the Hp was not significant (Figure 4C).

**Figure 5:**
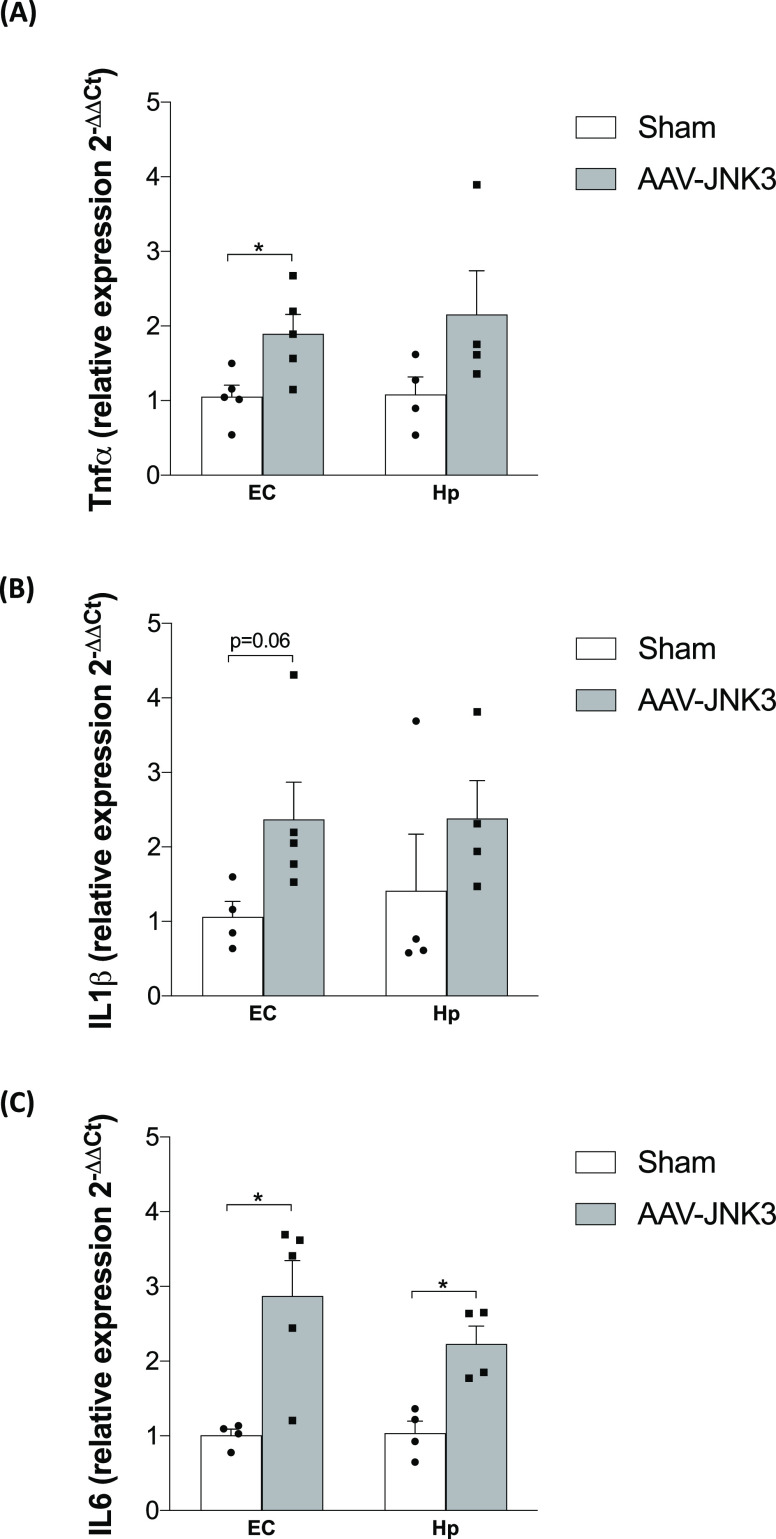
Effects of JNK3 overexpression on neuroinflammation. (A) TNFα mRNA relative expression in the EC (Student′s *t* test, *t* = 2.782, *p < 0.05; *n* = 5) and Hp (Student′s *t* test, *t* = 1.703, *p* > 0.05; *n* = 5). (B) IL-1β mRNA relative expression in the EC (Student′s *t* test, *t* = 2.207, *p* = 0.06; *n* = 5) and Hp (Student′s *t* test, *t* = 1.065, *p* > 0.05; *n* = 5). (C) IL-6 mRNA relative expression in the EC (Student′s *t* test, *t* = 3.445, *p < 0.05; *n* = 5) and Hp (Student′s *t* test, *t* = 4.123, *p < 0.05; *n* = 5). Results are shown as mean ± SEM and expressed as 2^-ΔΔCt^. EC: entorhinal cortex; Hp: hippocampus; TNFα: tumor necrosis factor α; IL-1β: interleukin 1β; and IL-6: interleukin 6.

In order to address the implication of JNK3 in the release of inflammatory mediators, proinflammatory cytokine (TNFα, IL-1β, and IL-6) mRNA expression was measured in both EC and Hp. All of the cytokines studied showed a marked increase not only in the EC but also in the Hp (Figure 5A–C); however, in the Hp, only the increase in IL-6 expression reached a statistical significance (Figure 5C), probably due to the high variability of the data obtained in TNFα and IL-1β (Figure 5A,B).

### 2.4 Effects of JNK3 Overexpression on Tau Pathology

JNK3 can be autophosphorylated and subsequently it can induce Tau hyperphosphorylation.^35^ In the present study, two different Tau conformations (ALZ50 and MC1) were analyzed, in an attempt to study the role of JNK3 in Tau aberrant misfolding.^40−45^ Moreover, tauopathy brains present truncated Tau forms that can adopt pathological conformations.^46^ Specifically, in the present study, Asp421 was the truncated form chosen, a form very prone to aggregation.^47,48^ Moreover, Tau Ser422 phosphorylation precedes Asp421 truncation.^49^ Therefore, this Tau phosphorylation (pTau Ser422) has also been assessed in this study.

**Figure 6:**
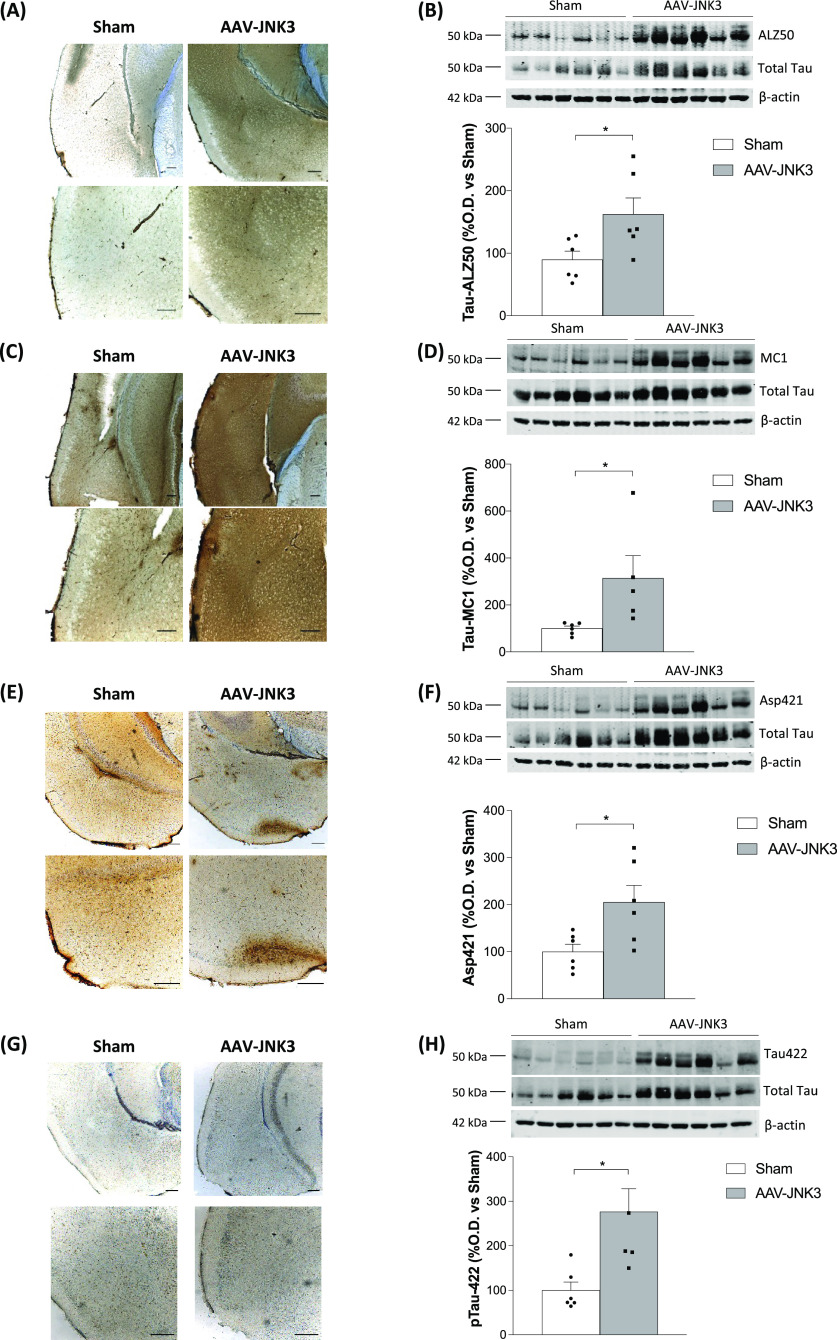
Effects of JNK3 overexpression on Tau in the EC. (A) Tau ALZ50 expression in Sham- and AAV-JNK3-injected (upper panels) mice and magnification images (lower panels) of EC. Scale bar: 200 μm. (B) Tau ALZ50 protein presence in the EC (Student′s *t* test, *t* = 2.459, *p < 0.05; *n* = 6). (C) Tau MC1 expression in Sham- and AAV-JNK3-injected mice (upper panels) and magnification images (lower panels) of EC. Scale bar: 200 μm. (D) Tau MC1 protein presence in the EC (Student′s *t* test, *t* = 2.458, *p < 0.05; *n* = 6). (E) Asp421-truncated Tau expression in Sham- and AAV-JNK3-injected mice (upper panels) and magnification images (lower panels) of EC. Scale bar: 200 μm. (F) Asp421-truncated Tau protein presence in the EC (Student′s *t* test, *t* = 2.694, *p < 0.05; *n* = 6). (G) pTau Ser422 expression in Sham- and AAV-JNK3-injected mice (upper panels) and magnification images (lower panels) of EC. Scale bar: 200 μm. (H) pTau Ser422 presence in the EC (Student′s *t* test, *t* = 3.211, *p < 0.05; *n* = 6). Results are shown as mean ± SEM. In panels (B), (D), (F) and (H), figures show optical density (O.D.) value percentage and an illustrative image of the blotting.

In the EC, AAV-JNK3-treated mice exhibited a strong increase on ALZ50 immunoreactivity (Figure 6A), which was further corroborated by an augmented signal in immunoblotting (Figure 6B). The same result was obtained for the other Tau conformational form, i.e., MC1 (Figure 6C,D). In the same line, truncated Asp421 (Figure 6E,F) and the preceding Ser422 phosphorylation (Figure 6G,H) also appeared to be significantly increased. Consistent with a post-transcriptional regulation of Tau, total protein levels, normalized using actin, remained unaltered.

**Figure 7:**
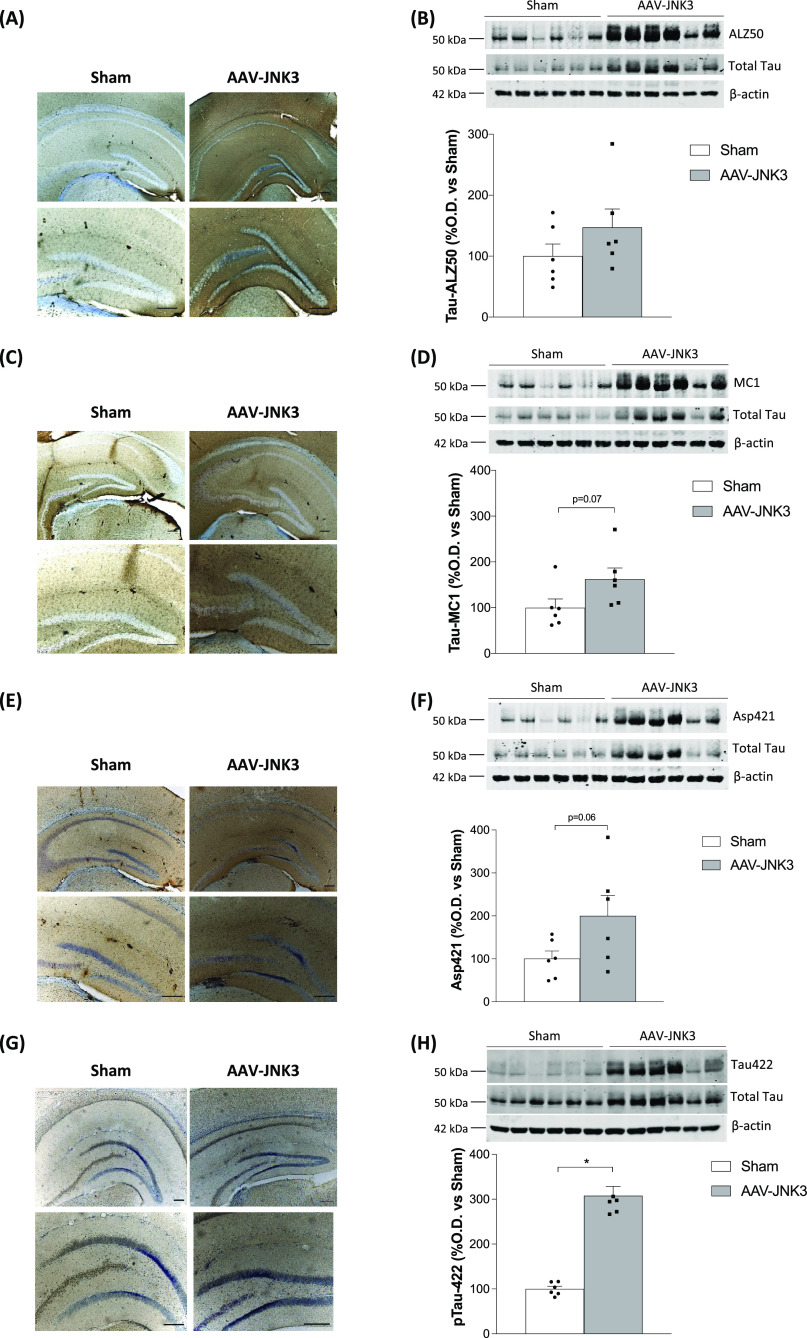
Effects of JNK3 overexpression on Tau in the Hp. (A) Tau ALZ50 expression in Sham- and AAV-JNK3-injected (upper panels) mice and magnification images (lower panels) of Hp. Scale bar: 200 μm. (B) Tau ALZ50 protein presence in the Hp (Student′s *t* test, *t* = 1.310, *p* > 0.05; *n* = 6). (C) Tau MC1 expression in Sham- and AAV-JNK3-injected mice (upper panels) and magnification images (lower panels) of Hp. Scale bar: 200 μm. (D) Tau MC1 protein presence in the Hp (Student′s *t* test, *t* = 2.011, *p* = 0.07; *n* = 6). (E) Asp421-truncated Tau expression in Sham- and AAV-JNK3-injected mice (upper panels) and magnification images (lower panels) of Hp. Scale bar: 200 μm. (F) Asp421-truncated Tau protein presence in the Hp (Student′s *t* test, *t* = 1.977, *p = 0.07; *n* = 6). (G) pTau Ser422 expression in Sham- and AAV-JNK3-injected mice (upper panels) and magnification images (lower panels) of Hp. Scale bar: 200 μm. (H) pTau Ser422 presence in the Hp (Student′s *t* test, *t* = 9.460, *p < 0.05; *n* = 6). Results are shown as mean ± SEM. In panels (B), (D), (F) and (H), figures show optical density (O.D.) value percentage and an illustrative image of the blotting.

The same conformational changes were studied in the Hp and although a marked immunostaining increase was observed in ALZ50 (Figure 7A,B), MC1 (Figure 7C,D), and Asp421 (Figure 7E,F), only pTau Ser422 reached significant increased levels (Figure 7G,H). Once again, total Tau protein levels, normalized using actin, remained unchanged.

## 3 Discussion

A broad variety of illnesses involve the JNK family.^50,51^ Indeed, JNKs are thought to be a critical mediator of neuronal response to stress, involving both neuronal survival and death under a variety of conditions.^52^ There are at least 10 JNK isoforms expressed from three genes, exhibiting differences in substrate and binding protein specificity.^6^ Knock-out animal models disclosed different gene product features,^9,16^ yet evidence for selective activation of endogenous JNKs is absent. Indeed, although many studies in the literature have addressed the cognitive and molecular consequences of JNK3 ablation in AD, to our knowledge, currently there is no study that analyzes the consequences of JNK3 overexpression on cognitive performance. Thus, the main aim of the present study was to assess the consequences of JNK overexpression, more specifically of the JNK3 isoform, i.e., the main isoform in the brain.

This work focuses on the EC as it is considered to be one of the key sites for the development of neurodegeneration. The EC is an essential area located in the medial temporal lobe, whose functions include long-term memory. Interestingly, the EC projects to the Hp and it receives inputs from other cortical areas. The EC is divided into two main areas: the medial EC (MEC) and the lateral EC (LEC). Both MEC and LEC has shown to have different functional characteristics. The MEC superficial layers comprise several spatially modulated cell types, whereas the LEC′s adjacent neurons exhibit only sparse spatial modulation^53−55^ and somatosensory information.^56−59^ The spatial information coming from the MEC together with the nonspatial information processed from the LEC is integrated in the EC.^60−63^ The EC is one of the earliest affected areas in neurodegenerative disorders such as AD, indicating the essential participation of EC in cognition.^64^ Although the reason behind this early EC impairment in AD is still unknown, a specific vulnerability to aging and AD of the EC neurons is hypothesized,^65^ which induces a significant neuronal death in this area during the first stages of the disease.^66^ Noteworthy, amyloid protein and hyperphosphorylated Tau aggregation, i.e., the main AD histopathological characteristics, appear first in the EC in mild AD and are not disseminated to other areas such as the Hp until more advanced stages of the disease.^67^ Hence, it has been suggested that the neurodegeneration that starts in EC neurons is transferred to the Hp, inducing the disruption of the cortical–hippocampal network in AD patients. In light of these important findings, in this study it was decided to induce JNK3 overexpression in both MEC and LEC, in order to elucidate if increased levels of JNK3 could lead to a cortical–hippocampal network dysfunction and ultimately to cognitive alterations. Furthermore, JNK3 overexpression was induced in wild-type mice, in an attempt to mimic early stages of AD when amyloid plaque or neurofibrillary tangle accumulations are still absent.

Our results showed that although viral infection was conducted in the EC (MEC and LEC), JNK3 overexpression is also observed in the Hp, concluding that changes in the EC can directly influence its main afferent areas, such as the Hp, leading to aberrant network activity as it has been observed in mouse models and human AD patients.^68,69^ More importantly, we demonstrated that JNK3 overexpression was associated with a behavioral impairment of associative memory, assessed by the NORT. A significant role in object recognition and novelty detection has already been assigned to the EC.^70^ In particular, information from EC can be acquired in the Hp through the intricate integration of spatial information coming from the MEC with nonspatial input from the LEC.^62,63^ Specifically, two LEC cell classes have been recognized, one of them firing at the objects and the other one firing at the places where the objects were located previously.^70^ In addition, the LEC is needed to recognize items encountered in a particular context^71^ and the specific lesion of the LEC impairs the capacity to distinguish either novel object-place or novel object-place-context associations.^71^ Therefore, in light of our results, it seems that the induction of JNK3 overexpression in the EC affects the integration of information in the Hp, leading to cognitive deficiencies. On the contrary, no alterations were observed in the MWM task after JNK3 overexpression. The MWM is a classical test to assess spatial and thus hippocampus-dependent memory performance.^72^ Therefore, our results suggest that the increase of JNK3 observed in the Hp is not strong enough to induce a spatial learning impairment, as it occurs in early stages of AD.

The proof that JNK accumulation is associated with inflammatory pathway activation^73^ proposes the main question of whether brain inflammation is involved in the early behavioral deficits found in the present study after JNK3 overexpression induction. Inflammation is the first reaction from our body′s immune system to pathogens or irritation and it is a two-edged sword. It protects tissue against invading agents under acute circumstances and encourages healing. On the other hand, it can cause severe damage to the host′s own tissue if it is chronically maintained. While the CNS is recognized as an immune-privileged organ, there is growing evidence that inflammation is directly involved in the pathogenesis of several neurodegenerative diseases, including AD, multiple sclerosis (MS), and HIV-associated dementia.^74−76^ Chronic inflammation-mediated tissue injury can be remarkably damaging to the brain, as neurons are usually irreplaceable. In particular, it has been extensively demonstrated the involvement of astrocytes and microglia in the pathological process of AD. Indeed, it has been observed in AD animal models and patients that the cognitive deficiencies are accompanied by chronic glial activation and proinflammatory cytokine production.^77^ Consequently, pathological markers indicative of astrogliosis and microgliosis are correlated with cognitive disturbances in AD.^78−80^ Increased levels of proinflammatory cytokines are detected in early phases of clinical AD patients and it is suggested that those cytokines contribute to the neurotoxicity observed in AD late stages.^81−84^ In agreement with those studies, our data demonstrated that overexpression of JNK3 induced all of the pathological markers observed in early stages of AD brains, i.e., microgliosis, astrogliosis, and proinflammatory cytokine (IL-1β, IL-6, TNFα) release that could contribute to the cognitive deficiencies observed in the JNK3-induced mice. Interestingly, although all of those markers were strongly increased in the EC (the injection area), neuroinflammation was milder in the Hp. This could also explain the absence of cognitive alterations in the MWM.

Neuroinflammatory response is tightly related to senile plaques.^2,85^ Therefore, it is tempting to speculate about the close link between increased JNK activation and Aβ levels in AD. Previous *in vitro* discoveries have revealed an increase in pJNK levels after treatment with Aβ in primary cortical and hippocampal cell cultures.^86−88^ Moreover, it has been described an elevated expression of pJNK in AD patients’ postmortem brain samples and a tight colocalization with Aβ.^89^ Furthermore, AD mouse models have shown that JNK activation is related with an increased burden of senile plaques.^31^ According to these data, our group has recently demonstrated that both Aβ and pJNK appear to be specifically increased in the familiar AD model Tg2576 and human AD samples while similar pJNK expression was found in other dementias (vascular dementia, Lewy body dementia, and frontotemporal dementia).^90^ Thus, it can be suggested that Aβ and JNK can induce a vicious circle that could result in neuroinflammation and contribute to neurodegeneration in AD.

Apart of its central role in neuroinflammation, JNK kinase can participate in AD pathology by its implication in Tau phosphorylation and subsequent neurofibrillary tangle formation.^91^ It has been demonstrated by *in vitro* experiments that a JNK3 isoform can be autophosphorylated and then, it can contribute to Tau hyperphosphorylation.^92^ Tau hyperphosphorylation induces its aberrant misfolding, followed by its dissociation from microtubules and aggregation in neurofibrillary tangles. In order to study the implication of JNK3 on the conformation of Tau aberrant misfolding, two different conformations were studied: ALZ50 and MC1. ALZ50 has been detected in brain homogenates^93^ inside susceptible neurons.^41,93−96^ MC1 appeared to be a good marker for early aggregation of Tau protein, before the appearance of neurofibrillary tangles.^97−99^ Truncation is another modification tightly associated with Tau deposition.^100,101^ Several studies in the literature propose that Tau truncation precedes Tau assembly^47,48,100−103^ and it has been associated not only with early but also advanced stages of AD.^104−107^ Of note, Tau Ser422 phosphorylation usually precedes Tau truncation. In our hands, all of the aberrant conformations studied (ALZ50, MC1, truncated Asp421 Tau, and pTau Ser422) appeared to be strongly increased after JNK3 overexpression, suggesting that Tau misfolding and subsequent microtubule disaggregation could be also underlying the cognitive deficiencies observed in AAV-JNK3 mice. Noteworthy, the fact that in the Hp Tau misfolding assessment did not reach a statistical significance might ground the lack of cognitive impairment in the MWM task.

In summary, the data obtained in the present study indicate that activation of inflammatory signals and induction of Tau *in vivo* misfolding triggered by an enriched JNK3 environment is a significant early event during the progressive EC dysfunction. Therefore, JNK3 overexpression can lead to the triggering of cognitive dysfunction resulting in the dissemination of neurodegeneration from EC to Hp and may be at the origin of the changes observed in early stages of AD.

## 4 Material and Methods

### 4.1 Animals

In this study, 12 weeks old ICR mice were used (Envigo, Huntingdon, UK). Animals were housed in a temperature- (21 ± 1 °C) and humidity (55 ± 1%)-controlled room on a 12 h light/dark cycle, with ad libitum access to a standard chow diet and water. Experimental procedures were conducted in accordance with the European and Spanish regulations (2003/65/EC; 1201/2005) for the care and use of laboratory animals and approved by the Ethical Committee of University of Navarra (ethical protocol number 038-17).

### 4.2 Cells

BHK-21 cells were cultured in BHK-21 Glasgow MEM (Gibco BRL) complemented with 5% FBS, 20 mM HEPES, 2 mM glutamine, 10% tryptose phosphate broth, 100 IU/mL penicillin, and 100 μg/mL streptomycin. HEK-293T and HuH-7 cells were cultured in DMEM (Gibco BRL) with 10% FBS, 2 mM glutamine, 100 U/mL penicillin, and 100 μg/mL streptomycin.

### 4.3 Plasmid

A synthetic gene containing the coding sequences of mouse JNK3 isoform (NCBI Reference Sequence: NP_001075036.1) and green fluorescent protein (GFP) linked by the IRES (internal ribosome entry site) sequence of the encephalomyocarditis virus was generated. The IRES sequence allows JNK3 and GFP to be translated from the same mRNA, which allows us to identify cells that express recombinant JNK3 *in vivo*. The synthesis of this gene was entrusted to the company GenScript (Piscataway). The synthetic cassette was subcloned into the pAAV-CAG-GFP plasmid (Pignataro et al.^108^), substituting the GFP gene. In this way, the plasmid pAAV-CAG-JNK3-GFP was generated in which the JNK3-IRES-GFP sequence is under the transcriptional control of the constitutive CAG promoter. This promoter is highly effective for expression in neurons.^108^

### 4.4 Viral Vector Production

HEK-293T cells were cotransfected with a plasmid-containing ITR-flanked transgene construct (pAAV-CAG-JNK3-GFP) and a plasmid containing the adenoviral helper gene AAV8 cap (named pDP8.ape, PlasmidFactory, Bielefeld, Germany), using linear polyethylenimine 25 kDa (Polysciences, Warrington, PA). Seventy-two hours post-transfection, the supernatant was treated with PEG8000 (8% v/v final concentration) for 48–72 h at 4 °C. Then, the supernatant was centrifuged at 1500*g* for 15 min. Cells that incorporated AAV particles were treated with lysis buffer (50 mM Tris-Cl, 2 mM MgCl_2_, 150 mM NaCl, 0.1% Triton X-100) and were mantained at −80 °C. Supernatant and cell lysate were subjected to three cycles of freezing and thawing. Viral particles were purified from cell supernatant and lysate by ultracentrifugation at 350 000*g* for 2.5 h in a 15–57% iodixanol gradient. The viral batches were then concentrated further by passage through Amicon Ultra Centrifugal Filters-Ultracel 100 K (Millipore, Burlington, MA). Vector stocks were stored at −80 °C.

For AAV vector titers (viral particles (vp)/ml) determination PCR for viral genome copies extracted from DNAase-treated viral particles (High Pure Viral Nucleic Acid Kit, Roche) was employed, using the following primers: forward-eGFP 5′-GTCCGCCCTGAGCAAACA-3′ and reverse-eGFP 5′ TCCAGCAGGACCATGTGATC-3′. More than 10^12^ viral genomes (VG)/ml vector titers were obtained.

### 4.5 Analysis of JNK3 Expression *In Vitro*

BHK and HuH-7 cells were transfected with 2, 4, and 6 μg of pAAV-CAG-JNK3-GFP plasmids using lipofectamine 2000 (Thermo Fisher). Cells were fixed at 24 h and pJNK and total JNK expression was studied by immunoblotting and immunofluorescence using an anti-pJNK primary mouse monoclonal antibody (1:1000, Cell Signaling Technology, Beverly, MA) and anti-totalJNK (1:1000, Cell Signaling Technology, Beverly, MA). A donkey anti-rabbit Alexa-546-conjugated antiserum (Invitrogen ref A21202, 1:1000) was used for detection.

### 4.6 Intraentorhinal Injection

Intraentrorhinal injection of JNK3-AAV (1 ×10^10^ vp) was stereotaxically performed in both lateral (LEC) (1 μL) and medial (MEC) (0.5 μL) entorhinal cortex (*n* = 14). The coordinates for targeting the LEC were anterior–posterior, −4.1; medial–lateral, ±4.3; and dorsoventral, −4.9 from the bregma. Coordinates for the MEC were anterior–posterior, −4.1; medial–lateral, ±3.5; and dorsoventral, −5.1 from the bregma. Sham animals (*n* = 12) received equivalent amounts of sterile PBS. Mice were sacrificed 3 months after the injection.

### 4.7 Behavioral Tests

All behavioral test were analyzed using a video-tracking system (Ethovision 11.5, Noldus Information Technology B.V., The Netherlands).

#### 4.7.1 Open Field Test

Animals were placed in an open field (35 × 35 cm^2^, 45 cm height) for 30 min and were allowed to explore in order to measure the spontaneous locomotor activity, in a dimly illuminated room.Total path distance (cm) was analyzed.

#### 4.7.2 Novel Object Recognition Test (NORT)

The procedure consists of three phases: habituation, training (sample phase), and test phase. The habituation phase was performed the previous day of the test day, in which each animal was allowed to freely explore the open field in the absence of objects. Next day, during the sample phase, a single animal was placed in the open field containing two identical objects for 5 min. After 1 h, the object of the right side was replaced for another one different enough to be easily discriminated by mice, but with similar degree of complexity. Animals were allowed to explore for 5 min constituting the test phase and the exploration time was recorded. Result was expressed as discrimination index and it is shown as percentage of time spent exploring the new object with respect to the total exploration time.

#### 4.7.3 Morris Water Maze (MWM)

The Morris water maze (MWM) was established in order to test hippocampal-dependent learning, including acquisition of spatial memory and long-term spatial memory. The MWM test is performed in a circular pool (145 cm in diameter) containing water (21–22 °C) which is virtually divided into quadrants. Around the pool, different visual clues are located. The test is divided into three phases: habituation, acquisition, and retention.

In the first place, a platform with a visible object is placed into the pool. The habituation or visible platform phase consists of six trials in which the mouse tries to reach the platform. Each trial is finished when the mouse reached the platform or after 60 s. If the mice do not reach the platform, they were guided and placed on the platform for 15 s.

The second phase, the hidden-platform phase, is performed during nine consecutive days. In this case, the platform is placed 1 cm below the water surface in a manner that it is hidden for the mice. Four trials per day are carried out; in each trial, mice were introduced to the pool from a different starting point. As in the habituation phase, the trial finished when the mouse reached the platform or after 60 s.

To test memory, probe trials were performed at the 4th, 7th, and last day of the test (10th day). In those probe tests, the platform was removed from the pool and mice were allowed to search it in the water for 60 s.

### 4.8 Biochemical Measurements

#### 4.8.1 Tissue Collection

Mice were euthanized by decapitation. Brains were immediately extracted and dissected on ice to obtain the EC and the hippocampus, and stored at −80 °C.

For immunohistochemistry studies, the right hemisphere was fixed by immersion in 4% paraformaldehyde in 0.1 M PBS (pH 7.4) for 72 h, followed by 30% sucrose solution. Brains were cut into series of 40 μm slices.

#### 4.8.2 Immunofluorescence (IF)

Brain sections were washed three times (10 min each) with PBS 0.1 M (pH 7.4) and incubated in blocking solution (PBS containing 0.1% BSA, 2% normal donkey serum, and 0.3% Triton X-100) for 2 h at room temperature. Incubation with the primary antibody (diluted in the blocking solution) was performed overnight at 4 °C. Then, sections were washed with PBS (3 × 10 min) and incubated with the secondary antibody (diluted in the blocking solution) for 2 h at room temperature, in the darkness. The primary antibodies used were anti-GFP (1:1000, Invitrogen, Carlsbad, California), anti-GFAP (1:1000, Cell Signaling Technology, Beverly, MA), and anti-Iba1 (1:1000, Wako, Osaka, Japan). Secondary antibody used was Alexa Fluor 546 goat anti-mouse for GFAP and Alexa Fluor 488 goat anti-rabbit for GFP and Iba1 (1:400, Invitrogen–Molecular Probes, Eugene, OR). To guarantee comparable immunostaining, sections were treated together under same conditions. Fluorescence signals were detected with a confocal microscope LSM 510 Meta (Carl Zeiss, Germany).

#### 4.8.3 Immunohistochemistry

Immunohistochemical examination of brains was performed using mouse monoclonal antibodies against Tau MC1 epitope (1:100, donated by Peter Davies, Department of Pathology, Albert Einstein College of Medicine), Tau ALZ50 epitope (1:100, donated by Peter Davies, Department of Pathology, Albert Einstein College of Medicine), Asp421-cleaved Tau clone C3 (1:250, Merck, Darmstadt, Germany), and Ser422 phospho-Tau (1:250, Thermo Fisher, Waltham, Massachusetts). Antibody binding was detected with a biotinylated secondary antibody, and the antibodies were visualized using an avidin–biotin–peroxidase complex with 3,3′-diaminobenzidine tetrahydrochloride (DAB) as the chromogen.

#### 4.8.4 Quantitative Polymerase Chain Reaction (qPCR)

For qPCR analysis, total RNA was extracted from respective tissues using Trizol reagent. Isolated total RNA was reverse-transcribed into cDNA using commercially available kits (Applied Biosystems). All subsequent qPCR reactions were performed on a QuantStudio 7 Flex Real-Time PCR System (Applied Biosystems). For normalization threshold cycles (Ct values) of all replicate, analyses were normalized to Gapdh within each sample to obtain sample-specific ΔCt values (=Ct gene of interest – Ct Gapdh). The following Taqman probes (Applied Biosystems) were used: MAPK10 (Mm00436518_m1), TNFα (Mm00443258_m1), IL-1β (Mm00434228_m1), and IL-6 (Mm00446190_m1).

#### 4.8.5 Western Blotting (WB)

Total protein homogenates were obtained by homogenizing the dissected EC or Hp in ice-cold lysis buffer (NaCl 200 mM, HEPES 100 mM, glycerol 10%, NaF 200mM, Na_4_P_2_O_7_ 2 mM, EDTA 5 mM, EGTA 1 mM, DTT 2 mM, PMSF 0.5 mM, orthovanadate 1 mM, and NP-40, inhibitors of proteases, and inhibitors of phosphatases at 1%) and centrifuged at 13 000 rpm 4 °C for 20 min. The supernatant was aliquoted and frozen at −80 °C until use. Homogenates (30 μg of protein) were separated by electrophoresis on polyacrylamide gels (7.5%). Membranes were probed overnight at 4 °C with the following primary antibodies: anti-GFAP (1:1000, Cell Signaling Technology, Beverly, MA), anti-CD11b (1:1000, NB110-89474, Minneapolis, MN), anti-pJNK (1:1000, Cell Signaling Technology, Beverly, MA), anti-totalJNK (1:1000, Cell Signaling Technology, Beverly, MA), Tau MC1 epitope (1:1000, donated by Peter Davies, Department of Pathology, Albert Einstein College of Medicine), Tau ALZ50 epitope (1:1000, donated by Peter Davies, Department of Pathology, Albert Einstein College of Medicine), Asp421-cleaved Tau clone C3 (1:1000, Merck, Darmstadt, Germany), and Ser422 phospho-Tau (1:1000, Thermo Fisher, Waltham, Massachusetts). Secondary antibodies conjugated to IRDye 800CW or IRDye 680CW (LI-COR Biosciences, Lincoln, NE) were diluted to 1:5000 in TBS with 5% BSA. Bands were visualized using the Odyssey Infrared Imaging System (LI-COR Biosciences, Lincoln, NE). Optical density (O.D.) was quantified for each band using Image Studio Lite software and normalized with β-actin (mouse monoclonal, 1:10000, Sigma-Aldrich) that was used as an internal control.

### 4.9 Statistical Analysis

Results, reported as means ± SEM, were analyzed by GraphPad Prism 6.0 and normality was checked by Shapiro–Wilk′s test (*p* < 0.05). The acquisition phase of the MWM was analyzed by two-way repeated-measures ANOVA. Data in the retention phase and neurochemical data were analyzed with Student′s *t* test. In all cases, the significance level was set at *p* < 0.05.

## Supporting Information Available

JNK3 expression in vitro. BHK and HuH-7 cells were transfected with 2, 4, and 6 μg of pAAV-CAG-JNK3-GFP plasmids and analyzed at 24 h (Figure S1). Magnification images showing somatic-like fluorescent shapes in the injection site at the EC and fiberlike fluorescent shapes are observed in the Hp (Figure S2) (PDF)
