# Supplementary material for: JNK3 Overexpression in the Entorhinal Cortex Impacts on the Hippocampus and Induces Cognitive Deficiencies and Tau Misfolding
Source: ACS Chem Neurosci. 2023 May 26;14(11):2074–88. doi: 10.1021/acschemneuro.3c00092 (PMC10251484; doi:10.1021/acschemneuro.3c00092)
Supplement: Supplementary file 1 — cn3c00092_si_001.pdf [file cn3c00092_si_001.pdf]

SUPPORTING INFORMATION

JNK3 overexpression in the entorhinal cortex impacts on the hippocampus and induces cognitive deficiencies and Tau misfolding

Carlos G. Ardanaz[1,2], Amaia Ezkurdia[1,2], Arantza Bejarano[1], Beatriz Echarte[1], Cristian Smerdou[2,3], Eva Martisova[3], Iván Martínez-Valbuena[2,4,5], María-Rosario Luquin[2,4,6], María J. Ramírez[1,2], Maite Solas[1,2*]

[1]Department of Pharmacology and Toxicology, University of Navarra, 31008 Pamplona, Spain; [2]IdISNA, Navarra Institute for Health Research, 31008 Pamplona, Spain; [3]Division of Gene Therapy and Regulation of Gene Expression, Cima Universidad de Navarra, 31008 Pamplona, Spain; [4]Neurosciences Division, Cima Universidad de Navarra, 31008 Pamplona, Spain; [5]Tanz Centre for Research in Neurodegenerative Diseases, University of Toronto, M5S 1A8 Toronto, Canada; [6]Neurology Department, Clinica Universidad de Navarra, 31008 Pamplona , Spain.

*Correspondence: msolaszu@unav.es (Maite Solas)

Department of Pharmacology and Toxicology

University of Navarra

Irunlarrea 1, 31011 Pamplona (Spain)

Telf.: 0034 948425600 (806648)

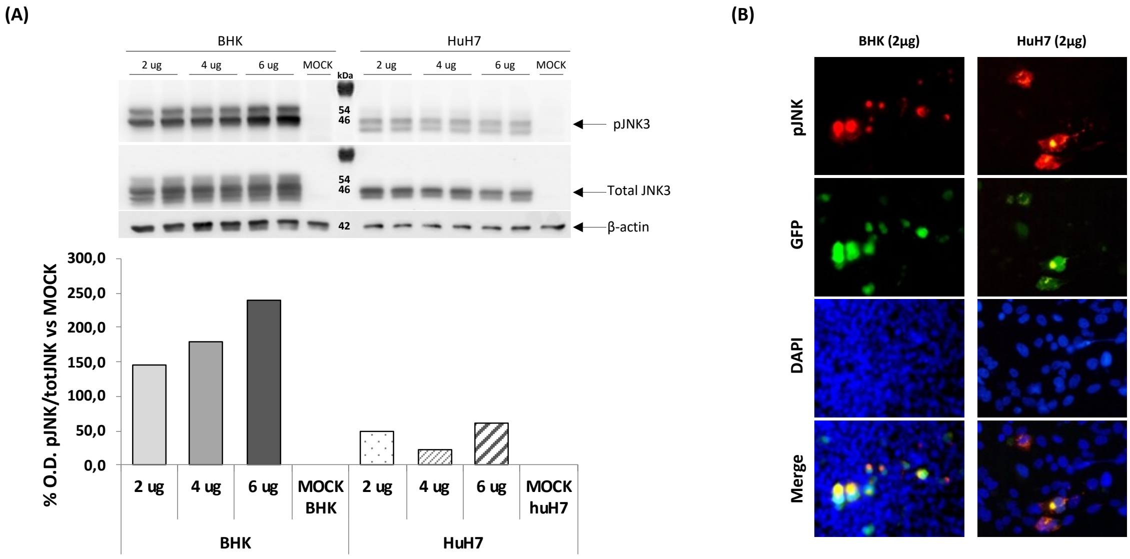

FIGURE S1. JNK3 expression in vitro. BHK and HuH-7 cells were transfected with 2, 4 and 6 μg of pAAV-CAG-JNK3-GFP plasmids and analysed at 24 h by (A) immunoblotting and (B) immunofluorescence with an antibody specific against JNK3. Results are shown as mean ± SEM. In panel A figures show optical density (O.D.) values percentage and an illustrative image of the blotting.

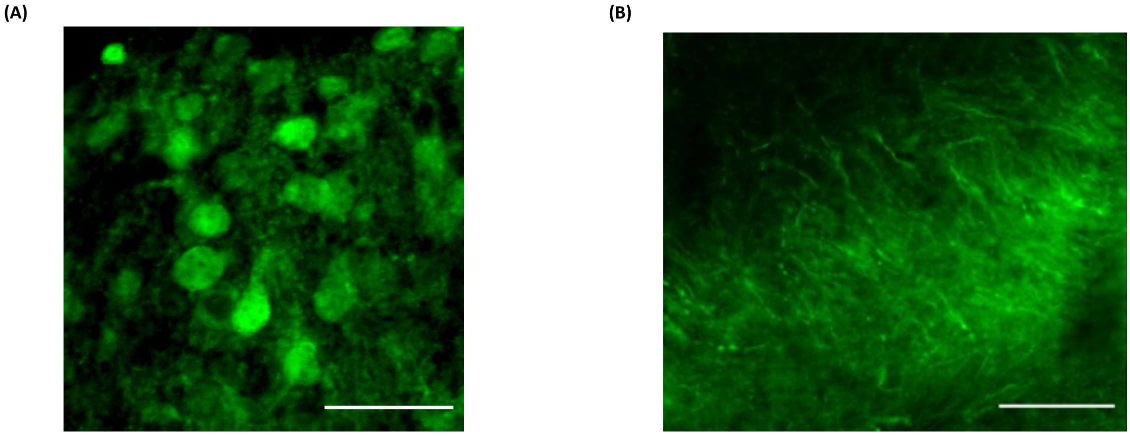

FIGURE S2. Magnification images showing somatic-like fluorescent shapes in the injection site at the EC (A) and fiber-like fluorescent shapes are observed in the Hp (B). Scale bar 50μm.
